# Sentinel lymph node biopsy using computed tomographic lymphography in patients with early tongue cancer

**DOI:** 10.3109/00016489.2015.1010126

**Published:** 2015-03-12

**Authors:** Kohei Honda, Koich Ishiyama, Shinsuke Suzuki, Eigo Oumi, Teruyuki Sato, Yohei Kawasaki, Hidekazu Saito, Kazuo Ishikawa

**Affiliations:** ^a^ ^1^Department of Otorhinolaryngology-Head and Neck Surgery; ^b^ ^2^Department of Radiology, Akita University Graduate School of Medicine, Akita, Japan

**Keywords:** Cervical lymph node, metastasis, iopamidol, blue dye method

## Abstract

*Conclusions:* Because computed tomography (CT) lymphography provides preoperative images of anatomic relationships between a tumor, its associated lymph vessels, and the sentinel lymph node (SLN), it may aid in directing the SLN biopsy for management of early tongue cancer. *Objectives:* SLN biopsy using a radioisotope (RI) generally has been performed in head and neck cancer. However, this method can be performed only at institutions that are licenced for its use. In this study, we evaluated the utility of performing SLN biopsy in patients with early tongue cancer using the newly developed technique of CT lymphography. *Methods:* Enrolled in this study were 31 patients with T1N0 or T2N0 tongue cancer. CT images were obtained before and after injection of iopamidol into the peritumoral region and the SLN was identified as the first enhanced lymph node. SLN biopsy was performed using CT lymphographic guidance combined with blue dye injection. *Results:* The SLN was detected by CT lymphography in 28 cases (90.3%). By intraoperative frozen section examination, metastases to SLNs were found in 4 (14.3%) (T1N0, 1 patient; T2N0, 3 patients) of the 28 patients. Of these four, SLN micrometastases were found in one patient.

## Introduction

Metastasis to the cervical lymph nodes represents the single most important prognostic indicator in head and neck squamous cell carcinoma (HNSCC), and management of the cervical lymph node is one of the most important factors in the control of HNSCC [1]. Although there have been advances in the use of imaging techniques such as computed tomography (CT), ultrasound, and positron emission tomography (PET), they are not sufficiently sensitive for detecting occult neck metastasis. Approximately 25–40% of patients diagnosed with T1N0 or T2N0 tongue cancer have occult metastases in the neck [2, 3, 4]. Therefore, if a ‘watchful waiting’ policy is chosen for T1/T2N0 tongue cancer, careful and frequent follow-up will be needed, which in turn causes both surgeons and patients to have much mental stress about neck recurrence after glossectomy. When neck recurrence is diagnosed, sometimes salvage neck dissection will be difficult because the tumor grows rapidly and results in late-stage regional failure. On the basis of the high incidence of neck recurrence, elective neck dissection has predominated until recently [5, 6]. However, the policy of elective neck dissection exposes at least 60% of patients with T1/T2N0 tongue cancer to an unnecessary procedure. Recently, the sentinel lymph node (SLN) concept has been introduced in HNSCC to detect neck metastases more precisely [7, 8]. The SLN is the first lymph node in the regional lymphatic basin to receive lymph draining from the primary neoplasm, and non-SLN metastases in the lymphatic basin are believed to be unlikely if the SLN is negative for metastasis [9]. SLN biopsy has become a standard surgical procedure for patients with N0 stage tongue cancer. The radioisotope (RI) and blue dye methods generally have been used to detect the SLN. However, the RI method can be performed only at licenced institutions by trained personnel. In addition, it is difficult to obtain images when the SLN is close to the RI injection site because of shine-through radioactivity. Recently, the use of CT lymphography, which provides images of the SLN and lymph vessels with surrounding anatomy, has been reported for breast cancer [10, 11]. However, to our knowledge, performance of SLN biopsy for tongue cancer using CT lymphography has not been reported. This study evaluated the usefulness of the SLN biopsy performed using CT lymphographic guidance combined with the blue dye method in patients with early tongue cancer.

## Material and methods

### Patients

During the period from April 2007 to November 2011, 31 patients (17 males and 14 females) with previously untreated early tongue cancer (squamous cell carcinoma) were enrolled in this study. They were 33–91 years old (mean age 64.0). Patients presented with a T1 or T2 primary lesion at clinical or radiologic stage N0 (T1N0, 14 patients; T2N0, 17 patients). The minimum follow-up period was 30 months. This study was approved by the Ethics Committee of Akita University School of Medicine, and informed consent was obtained from all patients.

### CT lymphography

**Figure 1.**
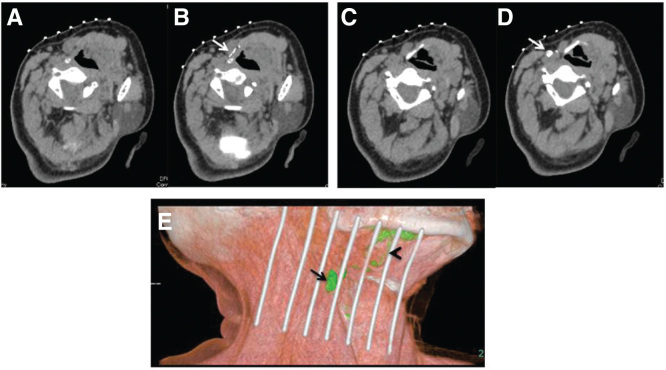
**A case of CT lymphography for T2N0 tongue cancer. (**A**) Control CT image before iopamidol injection (section at lymph vessel level). (**B**) CT image 1 min after iopamidol injection. Arrow, lymph vessel. (**C**) Control CT image before iopamidol injection (section at sentinel lymph node (SLN) level). (**D**) CT image 3 mins after iopamidol injection. Arrow, SLN, which is the first enhanced lymph node. (**E**) Three-dimensional CT lymphography reconstructed from the images after injection. Arrow, SLN; arrowhead, lymph vessel.**

**Figure 2.**
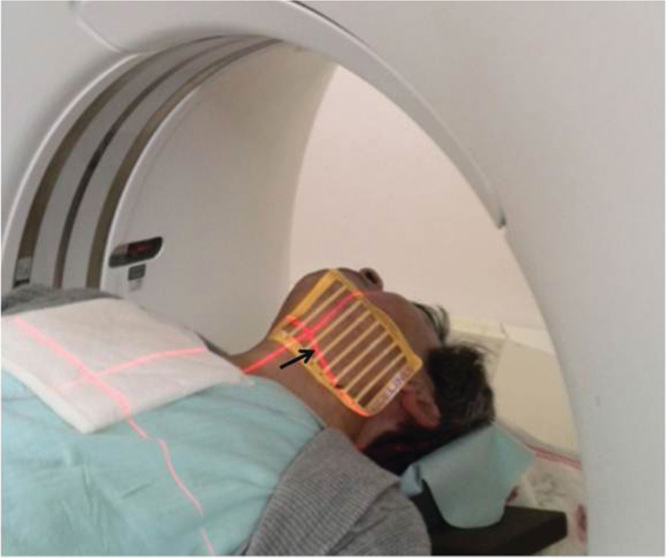
The sentinel lymph node (SLN) location was indicated precisely by the crossing points of the lattice marker and the CT plane light. Arrow, CT plane light.

CT lymphography was performed to identify the SLN on the day before the SLN biopsy and glossectomy. Each patient was placed in the same position as for the subsequent surgery, with the neck extended and inclined toward the healthy side. A lattice marker (CT Guidelines, FLAIR Co. Ltd, Tokyo, Japan), which is a commodity developed for the CT-guided needle biopsy, was attached to the skin at the neck. First, non-contrast CT images of the oral cavity and neck were obtained by using a 64 multi-detector row CT scanner (Discovery 750 HD, GE Healthcare, Milwaukee, WI, USA). CT scanning was performed with the following parameters: a tube voltage of 120 kVp, auto mA (noise index, 26.26), 0.969:1 beam pitch, standard reconstruction mode, 20 mm detector coverage, helical thickness of 0.625 mm, scan type of helical mode, and rotation time of 0.6 s. Next, 1.5 ml of iopamidol (Iopamidol 370; Bayer Healthcare, Osaka, Japan) and 0.5 ml of 1% lidocaine hydrochloride were mixed and injected into the peritumoral area with a 25-gauge needle. CT images were obtained 1, 3, 5, and 10 min after administration of the iopamidol. The CT images were analyzed at once and the SLN was identified as the first enhancing lymph node in the lymphatic flow from the injection site (Figure 1). The SLN location was indicated precisely by the crossing points of the lattice marker and the CT plane lights. This point was marked with an oil pen as the SLN (Figure 2).

### SLN biopsy and surgery

**Figure 3.**
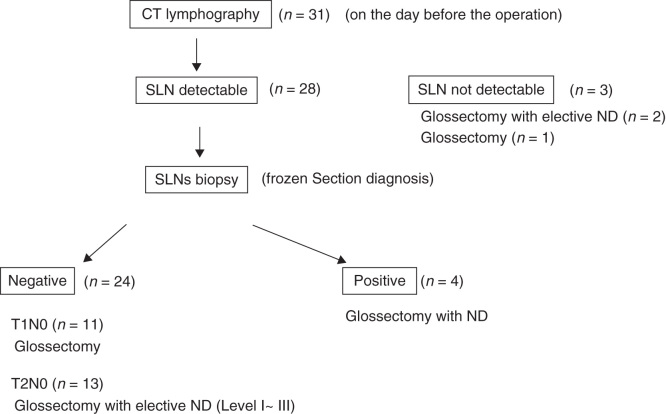
Therapeutic options and patient distribution: 31 patients with T1N0 or T2N0 tongue cancer were enrolled in this study. The day after the CT lymphography, sentinel lymph node (SLN) biopsy using CT lymphographic guidance combined with the blue dye method and glossectomy were performed in the 28 patients having identified SLNs with CT lymphography. When the SLN was negative for metastasis in T1N0 cases, only glossectomy without neck dissection (ND) was performed. For T2N0 cases, primary tumor resection with elective neck dissection (level I–III) was performed regardless of the presence of metastasis in the SLN.

SLN biopsy was performed under CT lymphographic guidance combined with patent blue dye injection. Just before SLN biopsy, 2 ml of 1% patent blue was injected into the peritumor area. The SLN biopsy was performed using a skin incision at the previous day’s SLN marker made in accordance with the CT lymphography images. The dyed node located just under this marker was defined as the SLN. When a blue dyed node was not detected, SLN biopsy was performed only by the CT lymphographic guidance. Intraoperative frozen section examination of the SLN was performed. When the SLN was negative for metastasis in T1N0 cases, only glossectomy without neck dissection was performed. For T2N0 cases, primary tumor resection with elective neck dissection (level I–III) was performed regardless of the presence of metastasis in the SLN, in order to check for non-SLN metastasis. With positive metastasis to the SLN, glossectomy and elective neck dissection were performed, guided by the area of lymph node involvement (Figure 3). When the SLN was not detected by the CT lymphography, elective neck dissection was recommended in the case of T2N0.

## Results

CT lymphography images of both enhanced lymph vessels draining the tumor injection site and SLNs were obtained in 28 patients (90.3%); lymph vessels only were detected in 1 patient (3.2%); and neither lymph vessels nor an SLN were detected in 2 patients (6.5%). The time to detection of the nodal enhancement was 1 min in 2 patients (7.1%), 3 min in 15 patients (53.6%), 5 min in 5 patients (17.9%), and 10 min in 6 patients (21.4%). The number of SLNs detected with CT lymphography was 0 in 3 patients (9.7%), 1 in 11 patients (35.5%), 2 in 11 patients (35.5%), and 3 in 6 patients (19.4%). Of the 28 patients, 16 patients (57.1%) had SLNs at level II, 7 patients (25.0%) at levels I and II, 3 patients (10.7%) at level I, and 2 patients (7.1%) at level III (Table I).

**Table I.** **Results of CT lymphography (*n* = 31).**

Characteristic	*n* (%)
*Findings of CT lymphography*	
LV(+), SLN(+)	28 (90.3)
LV(+), SLN(–)	1 (3.2)
LV(–), SLN(–)	2 (6.5)
*Time to nodal enhancement (min)*	
1	2 (7.1)
3	15 (53.6)
5	5 (17.9)
10	6 (21.4)
*No. of SLNs*	
0	3 (9.7)
1	11 (35.5)
2	11 (35.5)
3	6 (19.4)
*Level of SLN*	
I	3 (10.7)
II	16 (57.1)
III	2 (7.1)
1 and II	7 (25.0)

LV, lymph vessels; SLN, sentinel lymph node.

The day after the CT lymphography, SLN biopsy using CT lymphographic guidance combined with the blue dye method and glossectomy were performed in the 28 patients having identified SLNs with CT lymphography. Of these 28 patients, SLNs were also detected by the blue dye method in 20 patients (T1N0, 10 patients; T2N0, 10 patients) (66.7%). All SLNs detected by the blue dye method were also detected by CT lymphography. Metastases to SLNs were found by intraoperative frozen section examination in 4 (14.3%) (T1N0, 1 patient; T2N0, 3 patients) of the 28 patients (Table II). For the 13 T2N0 patients with negative SLNs, selective neck dissection (levels I–III) and glossectomy were performed and no node metastases were found. For the 11 T1N0 patients with negative SLNs, only glossectomy was performed without elective neck dissection. Among the four patients with metastases to the SLN, SLN micrometastases were found in one patient. In one case, modified radical neck dissection was performed because the SLNs in level II and other nodes in level IV were also found to have metastases by intraoperative frozen section examination. For the other three patients with SLN metastasis in level I or II, elective neck dissection at levels I–III was performed because non-SLNs in level IV were found to be negative for metastases. During the follow-up period (a minimum of 30 months), neck recurrence was found in only 1 patient (T1N0) of the 24 who initially had metastasis-negative SLNs.

**Table II.** **Results of SLN biopsies (*n* = 28).**

Characteristic	T1N0	T2N0	Total (%)
*Detection of SLN with blue dye*			
Yes	10	10	20 (66.7)
No	2	6	8 (26.7)
*SLN histopathologic status*			
Negative	11	13	24 (85.7)
Positive	1	3	4 (14.3)

SLN, sentinel lymph node.

## Discussion

In this study, we determined that CT lymphography could provide preoperative images identifying the precise location of SLNs and lymph vessels draining the tumor, along with surrounding anatomy, in the early stage of tongue cancer. The sentinel node concept has been introduced previously for malignant melanomas and breast cancer [12, 13]. SLN biopsy for head and neck cancer also has been reported [7, 8]. The RI method and RI method combined with the dye method are generally used to detect SLNs. The blue dye method is simple and requires no special equipment but has a disadvantage of lack of preoperative detection because SLN is detected by only the naked eye during operation and a large skin incision is needed for SLN biopsy. Therefore, SLN biopsy using a single dye method is no longer performed. In the present study, during the follow-up period (a minimum of 30 months), neck recurrence was found in only 1 patient (T1N0) of the 24 who initially had metastasis-negative SLNs. In this case, SLNs that CT lymphography detected were not detected by the blue dye method. In a meta-analysis of SLN biopsy using the RI method of 766 patients with HNSCC, the sensitivity and negative predictive value of the SLN biopsy for all head and neck tumors were 95% and 96%, respectively [14]. The RI method has the advantages of high sensitivity and negative predictive value in SLN biopsy. However, there are some disadvantages of SLN biopsy with the RI method. The RI method can be performed only at licenced institutions by trained personnel, and lymphoscintigraphy has poor spatial resolution and lacks accurate anatomic landmarks and geometry. In addition, it is difficult to obtain images when the SLN is close to the injection site, because of shine-through radioactivity. Moreover, intraoperative external gamma-probe counting requires some skill to accurately identify the SLN. Therefore, a newer, simpler method not requiring the use of RI has been anticipated. Recently, SLNs have been reported to be well identified using CT lymphography without RI in patients with breast cancer and superficial esophageal cancer [10, 11, 15, 16, 17, 18, 19]. In the present study, CT lymphography could detect SLNs close to the injection site. Tangoku et al. [11] reported that an SLN was detected in all 40 T1 and T2 breast cancer patients when using CT lymphography, whereas dye navigation failed in 7 with fatty axillae and in 2 with prior excisional biopsy. Takahashi et al. [17] also reported that the SLN identification rates in breast cancer patients were 96% with CT lymphography, 92% with the dye-guided method, and 99% using both methods combined. The identification rates with CT lymphography and the combined method were significantly lower in node-positive patients compared with node-negative patients. With the dye-guided method, the SLN identification rate was significantly lower in patients with a body mass index (BMI) of 25 or higher, whereas for CT lymphography and the combined method the SLN identification rate was not influenced by the BMI [17]. In the present study, an SLN was not detected in three patients (79, 82, and 91 years old, respectively) by CT lymphography; all were T2N0 and all had BMIs under 25. For two of these patients, elective neck dissection (level I–III) was performed (pN0), and a ‘watchful waiting’ policy was chosen for the other. In one of the patients that underwent selective neck dissection, neck recurrence was found in level IV after 7 months, and salvage neck dissection was performed. It was reported that SLN found in level IV was very rare (1%) [8]. However, it can be hypothesized that SLN metastasis existed in level IV because at the beginning of this study, CT scanning did not cover the lower neck (level IV). CT scanning covered the lower neck in this study after level IV recurrence was found. These results indicate that careful follow-up must be conducted when SLN is not detected with CT lymphography.

MR lymphangiography has also reportedly provided temporal and anatomic localization of the SLN after injection of carbon dye into the tongue of adult pigs in a single investigation; clinical application of this technique in the future is anticipated [20].

The CT lymphographic method combined with the blue dye method in SLN biopsy has some advantages in that this method is simple and can be performed at any institution equipped with a CT scanner. It is quite useful for obtaining accurate anatomic images of the SLN, lymph vessels, and tumor before SLN biopsy. Moreover, in the present study, this method had the advantage of high negative predictive value (95.8%) in SLN biopsy but the disadvantage of lower sensitivity value (90.3%) compared with the RI method (95%). At present, SLN biopsy is not routine in oral cancer and is performed in only some facilities, but the use of CT lymphography will greatly increase the performance of SLN biopsy for patients with early tongue cancer in facilities all over the world.

## References

[CIT0001] Ferlito A, Rinaldo A, Robbins KT, Leemans CR, Shah JP, Andersen PE (2003). Changing concepts in the surgical management of the cervical node metastasis. Oral Oncol.

[CIT0002] Ho CM, Lam KH, Wei WI, Lau SK, Lam LK (1992). Occult lymph node metastasis in small oral tongue cancers. Head Neck.

[CIT0003] Cunningham MJ, Johnson JT, Myers EN, Schramm VL, Thearle PB (1986). Cervical lymph node metastasis after local excision of early squamous cell carcinoma of the oral cavity. Am J Surg.

[CIT0004] Van den Brekel MW, Stel HV, Castelijns JA, Nauta JJ, van der Waal I, Valk J (1990). Cervical lymph node metastasis: assessment of radiologic criteria. Radiology.

[CIT0005] Yuen AP, Wei WI, Wong YM, Tang KC (1997). Elective neck dissection versus observation in the treatment of early oral tongue carcinoma. Head Neck.

[CIT0006] Kligerman J, Lima RA, Soares JR, Prado L, Dias FL, Freitas EQ (1994). Supraomohyoid neck dissection in the treatment of T1/T2 squamous cell carcinoma of oral cavity. Am J Surg.

[CIT0007] Hoft S, Maune S, Muhle C, Brenner W, Czech N, Kampen W-U (2004). Sentinel lymph-node biopsy in head and neck cancer. Br J Cancer.

[CIT0008] Alkureishi LW, Ross GL, MacDonald DG, Shoaib T, Gray H, Robertson G (2007). Sentinel node in head and neck cancer: use of size criterion to upstage the N0 neck in head and neck squamous cell carcinoma. Head Neck.

[CIT0009] Cabanas RM (2000). The concept of sentinel lymph node. Recent Results Cancer Res.

[CIT0010] Suga K, Ogasawara N, Okada M, Matsunaga N (2003). Interstitial CT lymphography-guided localization of breast sentinel lymph node: preliminary results. Surgery.

[CIT0011] Tangoku A, Yamamoto S, Suga K, Ueda K, Nagashima Y, Hida M (2004). Sentinel lymph node biopsy using computed tomography-lymphography in patients with breast cancer. Surgery.

[CIT0012] Morton DL, Wen DR, Wong JH, Economou JS, Cagle LA, Storm FK (1992). Technical details of intraoperative lymphatic mapping for early stage melanoma. Arch Surg.

[CIT0013] Giuliano AE, Kirgan DM, Guenther JM, Morton DL (1994). Lymphatic mapping and sentinel lymphadenectomy for breast cancer. Ann Surg.

[CIT0014] Thompson CF, St John MA, Lawson G, Grogan T, Elashoff D, Mendelsohn AH (2013). Diagnostic value of sentinel lymph node biopsy in head and neck cancer: a meta-analysis. Eur Arch Otorhinolaryngol.

[CIT0015] Yamamoto S, Maeda N, Tamesa M, Nagashima Y, Yoshimura K, Oka M (2012). Prospective ultrasonographic prediction of sentinel lymph node metastasis by real-time virtual sonography constructed with three-dimensional computed tomography-lymphography in breast cancer patients. Breast Cancer.

[CIT0016] Motomura K, Sumino H, Noguchi A, Horinouchi T, Nakanishi K (2013). Sentinel nodes identified by computed tomography-lymphography accurately stage the axilla in patients with breast cancer. BMC Med Imaging.

[CIT0017] Takahashi M, Sasa M, Hirose C, Hisaoka S, Taki M, Hirose T (2008). Clinical efficacy and problems with CT lymphography in identifying the sentinel node in breast cancer. World J Surg Oncol.

[CIT0018] Hayashi H, Tangoku A, Suga K, Shimizu K, Ueda K, Yoshino S (2006). CT lymphography-navigated sentinel lymph node biopsy in patients with superficial esophageal cancer. Surgery.

[CIT0019] Suga K, Shimizu K, Kawakami Y, Tangoku A, Zaki M, Matsunaga N (2005). Lymphatic drainage from esophagogastric tract: feasibility of endoscopic CT lymphography for direct visualization of pathways. Radiology.

[CIT0020] Nason RW, Torchia MG, Morales CM, Thliveris J (2005). Dynamic MR lymphangiography and carbon dye for sentinel lymph node detection: a solution for sentinel lymph node biopsy in mucosal head and neck cancer. Head Neck.

